# How to build a habitable planet: an interview with Charles H. Langmuir

**DOI:** 10.1093/nsr/nwae060

**Published:** 2024-02-21

**Authors:** Yi-Gang Xu

**Affiliations:** Guangzhou Institute of Geochemistry, Chinese Academy of Sciences

## Abstract

Earth is the only known habitable planet in the solar system. Understanding how Earth developed its unique habitability has been the frontier of Earth sciences and has become one of the main themes of current deep-space explorations. What are the decisive factors that led to a habitable planet? What is the role of solid Earth processes in the origin of life and in modulating the surface environment? Are Earth's habitability studies relevant to current challenges that human beings face? These questions have attracted the interest of both scientists and the public alike. *NSR* spoke to Prof. Charles H. Langmuir from Harvard University in the USA, who is a solid Earth geochemist who carries out research on diverse aspects of the plate tectonic geochemical cycle, including ocean ridges, convergent margins and intraplate volcanism. Prof. Langmuir is the author of the book *How to Build a Habitable Planet* (www.habitableplanet.org), one of the best Earth science books published in 2012.

## IMPORTANCE AND HISTORY OF EARTH HABITABILITY STUDIES

***NSR:*** Several years ago, you published a book with Wally Broecker, *How to Build a Habitable Planet*, which has been influential in the field and to the public. Can you share with us your intention behind writing such a book and how you went about it?

***Langmuir:*** Wally wrote the original book with that title in 1984. It was a little book of seven chapters but had the grand scope of beginning with the Big Bang and ending up with humankind. Wally handed off the teaching of that course to me at Columbia, and I loved the book, but realized I needed to add a lot of material on the solid Earth to the course, and that the book was becoming outdated.

As I began what was to be a small project, I came to realize there were a lot of missing elements. In addition to little discussion of the solid Earth, there was no geobiology, no Earth history—and of course the emerging field of exoplanets had not been included. That began a decade-long process where I had to learn about many subjects in which I had no expertise—such as the origin of life and Earth history. This was an amazing opportunity to think about the whole universe and place Earth and human beings in that large context. I began to see an emerging theme of planetary evolution as a universal process, and to look for underlying principles that would transcend the particular details of what happened on Earth, and might apply more generally, even perhaps throughout the universe.

***NSR*:** Why is the study of Earth's habitability important? Is such study relevant to the challenges that human beings are currently facing, such as global warming?

**Figure 1:**
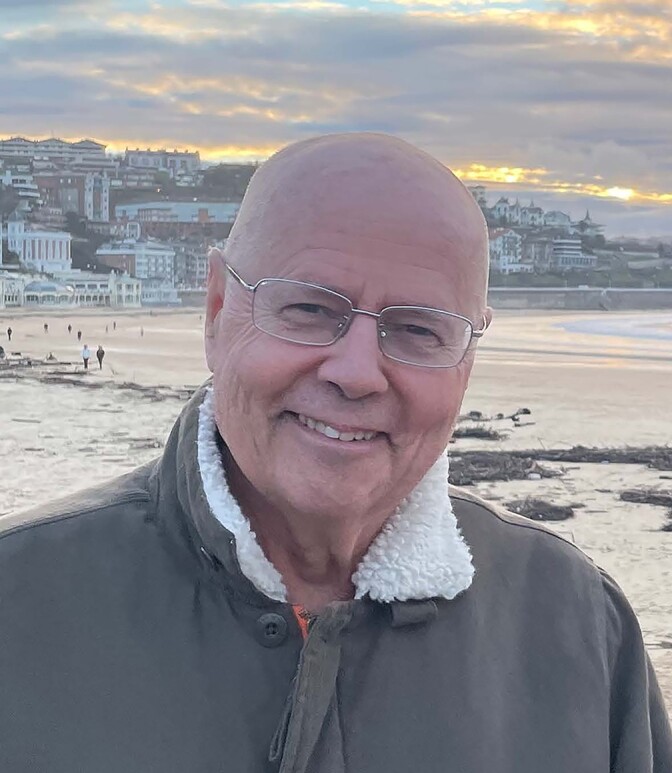
Prof. Charles H. Langmuir. (*Courtesy of Charles H. Langmuir*.)

***Langmuir:*** The common point of view is that global warming is the central issue we face, and that we had better do something about it because otherwise there will be negative consequences for our comfort and economic well-being. Viewed from that perspective, it becomes a problem more for economists and engineers—how much money is it worth spending to minimize global warming, and what might be the most economical engineering solutions? So, we need renewable energy and electric cars and redoing the electrical grid and so on. And there is the natural human tendency to want to ‘have our cake and eat it too’, and to want to fix things without pain or sacrifice. Doing something about global warming is fine in principle, but not if it impacts economic growth.

What if we were to solve CO_2_ emissions tomorrow? Would all our problems go away? No! The big problem is a much more general and widespread process of planetary destruction.

—Charles H. Langmuir

By working on the book I gradually came to a very different point of view. This becomes clear if we simply ask the hypothetical question, ‘What if we were to solve CO_2_ emissions tomorrow? Would all our problems go away?’ No! The big problem is a much more general and widespread process of planetary destruction. We are using up ground water, eroding soils and killing off other life on the planet at a scale and rapidity associated with mass extinctions of the past. As our population and economies grow, we cut down forests, build new cities, eat more fish and meat, inevitably kill vast numbers of other animals and plants and destroy planetary systems on which we and all other living creatures depend. In our current world, economic growth leads to planetary destruction, and that would continue unabated even if CO_2_ emissions went to zero.

For this reason I think we need a ‘planetary destruction index’ that would be reported as faithfully and thoroughly as GDP. Modification of the atmosphere would be one component, but others would be the abundance of other life, soil degradation, depletion of ground water, destruction of habitat, acidification of the oceans and human population. A moment of reflection shows that every component of this destruction index is increasing, and will continue to increase even if we drastically reduce greenhouse gas emissions.

That sounds rather bleak, but there is another aspect which comes from the long-term perspective of looking at Earth history as a process of planetary evolution. Human beings may have a positive role to play in this overall process. Earth started as a barren rock, and has gone through long periods in a rather stable configuration which then becomes radically modified as the planet evolves to a new state of functioning. For example, Earth was originally devoid of life and likely remained in that state for hundreds of millions of years. Earliest life was tiny prokaryotic cells that existed in the ocean with an atmosphere devoid of oxygen. This lasted for a billion years or so. The development of photosynthesis provided a source of oxygen, but oxygen was toxic to early organisms, and it took another billion years for life to protect itself from oxygen and even to use it as a mechanism to generate more energy from food. This greater energy potential led to much more complex unicellular organisms—the eukaryotic cells such as amoeba and paramecia. Ultimately Earth's surface was oxidized, O_2_ built up in the atmosphere, and multicellular organisms began a few hundred million years of development—invading the land, developing vertebrates, trees and so on. If one looks at these stages from a systemic point of view, rather than dwelling on the specifics, each one involved an increase in access to energy and an increasing scale of relationship.

Viewed from this systemic context, human civilization is a next stage of planetary evolution. We have had a revolution in access to energy, giving us domination over all of life and supporting super-exponential population growth, and an increasing scale of relationship across space and time, through language, printing and now all the various forms of electronic communication that create a global network that unites all of us instantaneously. There is much discussion of whether we have entered the ‘Anthropocene’ epoch—which is a very minor boundary in geological time. In the book we suggested instead that we are, potentially, at the beginning of an Anthropological Era, which in terms of its planetary effects is more pronounced even than any of the major changes in planetary function of the past. We have the ability to ponder the past, foresee the future and understand scales from the subatomic to the universal. And we have the capacity to undertake directed biological evolution, which is mind-boggling. No more is evolution dependent on random changes in DNA. These are the biggest changes in planetary function in 4.5 billion years, and they are just happening now with astonishing rapidity.

***NSR*:** What is the planetary perspective of Earth's habitability?

***Langmuir*:** As planets evolve over billions of years, the various step changes in planetary evolution are by no means assured. Most planets do not generate life, others may never pass the unicellular stage, and so on. Venus may have had early life, but it was too close to its star and had a runaway greenhouse. Mars may have had life, and perhaps may still in cracks beneath the surface, but it was too small and too far from its star. Other planets may not have accomplished the oxygen revolution, or been sterilized by a massive meteorite impact or a passing star. A female salmon lays thousands of eggs, and only a few survive to adulthood. There is jeopardy at every stage of development. Perhaps the universe is similar in that it spawns huge numbers of planets, and a very small proportion pass through all the stages of planetary evolution that we see on Earth.

From this perspective, we see the jeopardy of human civilization. We are in the process of planetary destruction, and it is by no means assured that our civilization will have longevity. All previous civilizations in human history have collapsed, but all of them were regional. None had the global consequences of our current world.

Global warming is then just one manifestation of this problem, and even for global warming we perceive it selfishly—how will it impact my life? How will it impact economic growth or jeopardize my living situation? We would need to live as

…we are, potentially, at the beginning of an Anthropological Era, which in terms of its planetary effects is more pronounced even than any of the major changes in planetary function of the past.

—Charles H. Langmuir

planetary protectors and sustainers rather than planetary users, and that would require an entirely new model of personal and societal behavior.

Perhaps studying Earth's history and evolution brings up these questions, which are both planetary and personal, and helps us to appreciate where we come from, to feel gratitude towards this planet and universe that have given rise to us and provide everything we need. And when we feel gratitude, we may begin to feel responsibility, and, if we really felt that, it might change our entire relationship to planetary systems. So why is the study of Earth's history and evolution important? Because it provides this very large context and perspective that pertain to the sense and aim of human existence.

## FACTORS THAT CONTRIBUTED TO THE EARTH'S HABITABILITY

***NSR***: When we look at the question of habitability in general, and examine Earth in that context, we see many very specific aspects of Earth that have contributed to its habitability. What does it take to make a planet habitable, and how common are those conditions likely to be?

***Langmuir*:** One fundamental requirement for habitability is climate stability, and that depends on the planetary volatile budget, particularly CO_2_ and H_2_O. It turns out the three elements in those molecules also make up more than 90% of the mass of living organisms. Those three elements are the most common elements in the galaxy apart from the non-reactive He. So, the universe makes in abundance the molecules needed for climate stability and life. Volatiles, however, are not efficiently accreted on rocky planets like Earth, so their overall abundance on Earth is small compared to other elements. Fortunately, they dissolve in magma at depth, and degas at the surface, so the planetary volatile budget is concentrated in surface reservoirs. But many factors could lead to highly variable volatile budgets on Earth-like planets.

How important are the exact amounts of volatiles? On Earth, water at the surface almost exactly fills the ocean basins, and the coexistence of oceans and continents provides long term climate stability. Is this a lucky accident or the product of feedbacks we do not yet understand? Do planets with more water become a ‘water world’ and can such a world have climate stability and life? The exact concentration of CO_2_ in the atmosphere is central to planetary temperature. We have some understanding of climate feedbacks on atmospheric CO_2_, but what would happen if a planet had 10 times less or more CO_2_?

The abundance of O_2_ is central to Earth’s ecosystems today and it appears to have been sustained in a narrow range for hundreds of millions of years. What feedbacks control atmospheric O_2_, how have they operated over planetary history, and how might they vary from one planet to another? A critical aspect that is often overlooked is the important role of the solid Earth. Plate tectonics and volcanism are the ultimate control on surface volatile abundance, and the evolution of O_2_ in the atmosphere is critically dependent not only on the source of O_2_ through photosynthesis and the burial of organic carbon, but also the sinks for O_2_ most of which are in the solid Earth through oxidation and reduction of Fe and S.

The question of universal habitability throws a different light on these questions. For example, is there some kind of feedback that makes planetary surfaces have just the right amount of water? Is a change from a reduced to oxidized surface likely to be a general consequence of planetary life? The operation of plate tectonics and its efficiency in connecting planetary layers would seem to be critically dependent on the mass of the planet. Must planets not only be within the habitable zone of their star, but also be in a habitable range of size in order for the volatile budgets to be sustained appropriately? These questions are only beginning to be investigated, and we are stuck with idealized models that may not give us reliable answers.

For planetary evolution there might be guiding principles that transcend the specifics and apply to all planets across a large range of planetary environments. The first is that habitability breeds habitability. If life destroys its environment, it disappears. Living systems that create an increasingly habitable environment will thrive. The second is that for life, increasing access to energy provides an evolutionary advantage. In that case, life will evolve to make use of more and more energy, just as has happened on Earth. The third is the advantage of symbioses, feedbacks and scale of relationship. Systems that are stable acquire longevity, and therefore succeed in the process of biological evolution. How these principles manifest in specific biological expression and planetary transformation may differ greatly from one planet to another, but would not the principles be universal? Finally, if Earth is a guide, over time planets make treasure—soils, groundwater, ore deposits, fossil fuels. This treasure then lies in waiting for a form of life that is able to make use of it. If these principles are generally applicable, might not intelligent civilizations be an eventual planetary outcome, barring catastrophic events?

***NSR***: What would be the most critical discovery that could address these issues?

***Langmuir:*** I am hardly alone in thinking that the most important question is how common life is in the galaxy. Is life ‘what planets do’ or is it exceedingly rare? That question will be definitively answered if we find life anywhere else. The arguments that life is rare, and Earth is possibly unique, means that life is statistically highly improbable. How can you make highly organized life out of a random collection of molecules? But there are

We would need to live as planetary protectors and sustainers rather than planetary users, and that would require an entirely new model of personal and societal behavior.

—Charles H. Langmuir

So, if we find evidence of life in one other place—be it Mars or Europa or a planet in another solar system—it means that life is overwhelmingly abundant.

—Charles H. Langmuir

100–200 billion stars in the Milky Way, and astronomers now tell us that most stars have planets, so there are roughly a trillion opportunities. If life is even a one in a million event, there would still be a million planets with life in the galaxy. The chance we would stumble across one of them, however, is one in a million. Astronomers have thus far found only a few thousand other planets, so we would need to look for many, many generations. So, if we find evidence of life in one other place—be it Mars or Europa or a planet in another solar system—it means that life is overwhelmingly abundant. It is not a statistical accident, but a property of the universe.

The other seminal discovery would be the ability to generate life in the laboratory. There is amazing progress being made in that respect. Human time is so short, and our imaginations so limited compared to the diversity of environments that may have existed early in Earth history, that if we can create laboratory conditions that engender life, then the origin of life is easy. Once you create an initial organism, then biological evolution over time leads to the amazing diversity of life. Make one simple proto-cell. If we can do that, on a miniscule human time scale, then again life is likely to be everywhere.

I would love to be alive when either of those discoveries happen, as it will be a pivotal moment in human history—we would realize with certainty that we are not alone; we are part of a living universe.

## MESSAGES TO YOUNG GENERATIONS OF RESEARCHERS

***NSR***: Could you give some advice to students and young scientists in the field of volatile circulation and Earth habitability?

***Langmuir:*** I am often asked in China by younger scientists, ‘How can I get a paper published in *Nature* or *Science*?’ because a publication in those journals unfortunately can be determinative for a young person's career. The way you get a paper published in these top journals is by asking good questions, by loving what you do, pursuing questions to completion and being open to discovery. And if a discovery happens, then if you are lucky and get a reasonable editor and reviewers, you can publish it in *Nature*. You can't manufacture it. In my career, most of my important papers have not been published in *Nature* and *Science*. In China the emphasis on where something is published is overdone—the emphasis should be on paper quality.

One of the features of the great scientists is that they look at the world with open eyes, and then question what they see. They then may discover new things that others have simply overlooked. If you can find the right question, and pursue it to completion, people often react with, ‘That's so obvious. How could I not have known that before?’ My great uncle won the Nobel Prize in chemistry, but one of his most important discoveries was in the field of physical oceanography. He loved to be outdoors even in bad weather, and one day when he was crossing the Atlantic on a ship in a raging storm he went out on deck to experience the wind and waves. He looked at the water and saw that seaweed arranged itself in equally spaced lines parallel to the wind. When the wind changed direction, the alignment changed within half an hour. This would have been apparent to sailors for perhaps a thousand years, but he saw it, and he asked himself, how could that happen? So, he later went to his summer cottage by a lake, and when a storm raged he went out in his boat and submerged umbrellas, and saw the umbrellas rotate in the water indicating convection cells with axes parallel to the wind. He was able to define the circulation and even measure velocities. ‘Langmuir circulations’ are now recognized as the primary way the shallow ocean mixes, and are central to the field of physical oceanography and an ongoing field of research.

Another time when he was in his boat on a calm day he noticed that a little drop of oil from the engine spread out into a big layer on the water. He asked, ‘I wonder how thick that layer is’, made measurements, and discovered mono-molecular films, with a journal in surface chemistry now named after him.

He did not make these discoveries by wondering how he could get a paper published in *Science* or *Nature*. He made it by being curious about the world, and following through on his curiosity with clever experiments and publications. Later in life he said that over his entire career he was just having fun.

When I was a boy my father carried on this tradition by doing fun science experiments with me—putting a candle in a milk bottle and then having the vacuum suck in a hard-boiled egg, floating bubbles in a tank of CO_2_, speaking in a squeaky voice by inhaling helium, building simple instruments. I learned that science was hands on, and fun. I have been very fortunate in my career that I too have been able to have fun doing what I love. I've never worried about getting awards or getting rich through science or any of these things. There was some luck involved—I was fortunate to enter the field of volcano geochemistry when there was very little data and the oceans were very little explored. Of course, in the current system I have to write proposals with

There are some other aspects that I consider very important as I look back on my career. First, get very good data … Second, be sure to model the data quantitatively and consider all the data … Finally, even though science can be competitive, relationships are more important.

—Charles H. Langmuir

hypotheses and so on, as if creative science could be engineered, but when the ship sets out to sea, or new data appear, I try to open my eyes and let discovery happen. And every once in a while, it does.

There are some other aspects that I consider very important as I look back on my career. First, get very good data. Question the quality of the data and work hard to make sure the data are right. Good data last forever. Second, be sure to model the data quantitatively and consider all the data, not just your own analyses of some isotope ratio or other. If you do not do that, you end up proposing ideas that do not work and will not stand the test of time. Finally, even though science can be competitive, relationships are more important. If you are at the end of your career and you have lots of important papers but no friends, you will not feel satisfied. So be generous—with one's samples, towards students, post-docs and the community. Science is a community activity—progress is made through a network of relationships, and we need to cultivate those.

## Author Biography

Yi-Gang Xu, Professor at the Guangzhou Institute of Geochemistry, Chinese Academy of Sciences, is an Editorial Board member of NSR and Guest Editor of the Special Topic.

